# Growth Development of French Children Born after *In Vitro* Maturation

**DOI:** 10.1371/journal.pone.0089713

**Published:** 2014-02-26

**Authors:** Laurence Foix-L’Hélias, Michael Grynberg, Béatrice Ducot, Nelly Frydman, Violaine Kerbrat, Jean Bouyer, Philippe Labrune

**Affiliations:** 1 Epidemiological Research Unit on Perinatal Health and Women’s and Children’s Health, Unité Mixte de Recherche S953, INSERM, Paris, France; 2 Service de Néonatologie, Hôpital Trousseau, Assistance Publique – Hôpitaux de Paris and Université Pierre et Marie Curie, Paris, France; 3 Service de Gynécologie-Obstétrique et Médecine de la Reproduction, Hôpital Antoine Béclère, Hôpitaux Universitaires Paris Sud, Assistance Publique – Hôpitaux de Paris, Clamart, France; 4 U782, INSERM, Clamart, France; 5 CESP Centre for Research in Epidemiology and Population Health, U1018, Reproduction and Child Development, INSERM, Villejuif, France; 6 UMRS 1018, Université Paris-Sud, Villejuif, France; 7 Unité de Formation et de Recherche Kremlin Bicêtre, Université Paris-Sud, Le Kremlin Bicêtre, France; 8 Service de Biologie de la Reproduction, Hôpital Antoine Béclère, Hôpitaux Universitaires Paris Sud, Assistance Publique – Hôpitaux de Paris, Clamart, France; 9 Service de Pédiatrie, Hôpital Antoine Béclère, Hôpitaux Universitaires Paris Sud, Assistance Publique – Hôpitaux de Paris, Clamart, France; INRA, France

## Abstract

**Background:**

Several lines of evidence indicate that immature oocyte retrieval and subsequent *in vitro* maturation (IVM) without ovarian stimulation may be a reliable option in assisted reproductive technologies (ART). However, few outcome data are available for children born following this technique.

**Objective:**

We assessed height and weight development of French children conceived after IVM.

**Methods:**

All children conceived after IVM at Antoine Beclere Hospital (Clamart, France) and born between June 2003 and October 2008 (*n* = 38) were included in a prospective cohort study and compared with a control group of children conceived by ICSI without IVM, matched for maternal age, gestational age and singleton/twin pregnancies. Follow-up included clinical examination at one year and a questionnaire completed by parents when the children were two years old (97% follow-up rate).

**Results:**

No statistical differences between IVM and control groups were found for boys. Mean weight, height and head circumference at birth were significantly greater for IVM than for ICSI girls (3.236 kg vs 2.701 kg (*p* = 0.03); 49 cm vs 47 cm (*p* = 0.01) and 34 cm vs 33 cm (*p* = 0.04), respectively). At one year, IVM girls remained heavier (mean weight 10.2 kg vs 8.6 kg (*p* = 0.001)) and taller (76 cm vs 73 cm (*p = *0.03)), and there was a two-point difference in BMI between the two groups of girls (18 vs 16 (*p* = 0.01)).

**Conclusion:**

Our results in girls born after IVM should be interpreted with caution. It remains unclear whether the observed sexual dimorphism is due to IVM technology or to maternal characteristics such as underlying infertility in patients with polycystic ovary syndrome (PCOS). Further monitoring of the outcomes of these infants is required.

## Introduction

In last decades, *in vitro* fertilisation (IVF) has become established as a reliable therapeutic option for overcoming infertility, resulting in the birth of more than four million children. Modern IVF protocols use high doses of exogenous gonadotropins to generate mature follicles. However, some women are extremely sensitive to controlled ovarian stimulation and are at risk of developing ovarian hyperstimulation syndrome (OHSS). The retrieval of immature oocytes from small antral follicles and their subsequent *in vitro* maturation (IVM) has therefore been proposed as an alternative option, to avoid the possible side effects of exogenous gonadotropin administration. Therefore, patients suffering from polycystic ovarian syndrome (PCOS) currently remain the main candidates for such a treatment [1]. However, the indications for IVM treatment have remarkably expanded, and now include poor responders to controlled ovarian hyperstimulation, and oocyte donation. The results of most studies investigating the health risks of children conceived after ICSI are reassuring [2] [3], but very little is known about the outcome of infants born after IVM, because this technique is much more recent [4]. Indeed, most studies reporting the outcome of infants conceived after IVM included only small numbers of patients, without controls [5] [6] [7] [8] [9]. However, theoretical concerns have been raised concerning the safety of IVM, including the risk of chromosomal abnormalities in the resulting embryos [10], the possible impact on the expression of imprinting genes [11] [12] and more recently a higher birth weight of IVM babies compared to ICSI babies [13]. To-date, this difference in birth weight has not been studied according to sex.

The objective of this study was to assess height and weight developments of a French cohort of children conceived after IVM.

## Materials and Methods

All infants conceived after IVM at Antoine Beclere Hospital (Clamart, France) and born between June 2003 and October 2008 (“IVM group”, *n* = 38) were included in a prospective cohort study. All mothers of these infants had PCOS. PCOS was characterised by endocrine disorders, with oligo- or anovulation, hyperandrogenism and polycystic ovaries.

These infants were compared with a control group of children conceived after ICSI without IVM (control group, mostly born to couples in whom the man displayed severe infertility) at the same centre and born between 2002 and 2008, matched for maternal age (≤35, >35), gestational age (<37, ≥37 WG) and singleton or twin pregnancies.

IVM and control embryos were generated during the study period at the same perinatal centre, by the same clinical and biological teams, and follow-up was undertaken by the same two paediatricians for both groups of patients.

### Definition of Treatments

#### IVM group

IVM was performed only in patients diagnosed with PCOS. In all cases the IVM protocol was performed as previously described [14]. Patients underwent a baseline ultrasound scan between days 2 and 4 of either a natural menstrual cycle, or, if amenorrheic, after withdrawal bleeding induced by progesterone treatment. to determine the number and size of antral follicles and endometrial thickness. The same evaluation was repeated between days 6 and 8 and when endometrial thickness was ≥6 mm, patients received 10,000 IU hCG i.m. Oocyte cumulus complexes (OCCs) were retrieved 35–36 hours after hCG administration, by trans-vaginal ultrasound-guided aspiration. Follicular fluids were collected in tubes containing 5 ml of prewarmed heparinised saline (100 µl in 250 ml NaCl). OCCs were washed in IVF medium (Origio, France) and matured in 1 ml of IVM medium (Origio, France) supplemented with 20% heat-inactivated serum from the patient, 0.75 IU/ml follicle-stimulating hormone (FSH) and 0.75 IU/ml luteinising hormone (LH) for 24 h. The oocytes were then denuded of cumulus cells with hyaluronidase and mechanical pipetting and their maturity was checked. Matured oocytes were inseminated by ICSI, with the partner’s spermatozoa.

#### Control group

Women underwent controlled ovarian hyperstimulation according to long agonist or antagonist protocols. In the long agonist protocol, patients received a time-release GnRH agonist (GnRH-a) on cycle day 2. Three weeks later, complete pituitary desensitisation was confirmed by the detection of low serum oestradiol (E_2_) and gonadotropin concentrations. Thereafter, recombinant FSH administration was initiated. Patients undergoing an antagonist protocol received micronised 17β-E_2_ oral tablets from day 20 of the cycle at treatment initiation until day 2 of the next cycle. On the first day of 17β-E_2_ discontinuation (cycle day 3), recombinant FSH administration was started. In both protocols, doses were adjusted according to the usual follicular maturation criteria. In antagonist protocols, GnRH antagonist was administered daily from day 6. We administered hCG (10,000 IU, i.m) when the follicles exceeded 17 mm in diameter and E_2_ concentration in the mature follicle exceeded 300 pg/ml. Oocytes were retrieved 36 hours after hCG administration, by trans-vaginal ultrasound-guided aspiration, and were inseminated by ICSI with the partner’s spermatozoa.

#### IVM and control groups

Fertilisation was assessed 17–19 hours after insemination, by checking for the appearance of two distinct pronuclei and two polar bodies. The zygotes were cultured in ISM1–ISM2 Medium (Origio, France). Embryos development was assessed on day 2 (41–43 h) and on day 3 (65–67 h) after insemination, by evaluating the regularity of blastomeres, the percentage and pattern of anucleate fragments and the dysmorphic characteristics of the embryos. The best embryos were transferred on day 2 or day 3 after ICSI.

We collected the following data for the mother’s characteristics: maternal age, parity, smoking status, pre-pregnancy body mass index (BMI defined as the ratio (weight (kg)/height^2^ (m^2^)). These data were extracted from hospital medical records. We also collected maternal information about infertility treatment, delivery and health of the infant at birth.

Maternal age was considered as a quantitative variable; tobacco use was considered as a dichotomous variable, with the categories “smoker” and “non-smoker” during pregnancy.

All women had undergone screening for gestational diabetes by a two-step approach [15]. An initial screening was performed between 24 and 28 weeks of gestation, by determining the plasma or serum concentration of glucose, one hour after a oral loading with 50 g of glucose (glucose challenge test (GCT)). In women whose glucose concentration exceeding the threshold value for the GCT (7.8 mmol/l), a diagnostic oral glucose tolerance test (OGTT) was performed. for which the diagnostic criteria for the OGTT (oral loading with 100 g glucose) were as follows: gestational diabetes mellitus was diagnosed if two or more of the blood glucose concentrations obtained reached or exceeded the threshold values of 5.3 mmol/l after an overnight fast, 10.0 mmol/l after 1 hour, 8.6 mmol/l after 2 hours and 7.8 mmol/l after 3 hours.

We recorded the following characteristics of the neonates: sex, birth weight, height, head circumference, APGAR score. All these data were obtained from hospital records. Follow-up involved clinical examination at the age of one year and a questionnaire completed by the parents when the child was two years old. Parents provided data on weight, height and head circumference based on the results of the clinical examination conducted at 2 years of age and reported in their child’s health booklet.

This study was approved by the C.C.T.I.R.S. (Comité consultatif sur le traitement de l’information en matière de recherche – National consultative committee on the treatment of information for research) and registered under number “06.192”. Parents were told about the study by medical staff during the pregnancy and were provided with written information. Written informed consent was collected from the parents for themselves and on behalf of their children involved in the study by the paediatrician during the clinical examination at the age of one year.

IVM and ICSI groups were compared using Student’s *t* tests for quantitative variables and chi-squared tests for qualitative variables. Non-parametric Kruskal-Wallis tests were used to compare IVM and ICSI children by sex. Statistical tests were two-tailed, with *p*<0.05 considered significant.

## Results

The study included a total of 76 infants born from 68 pregnancies (60 singletons and 8 sets of twins); 38 infants were born following IVM, and the control group also included 38 infants. The characteristics of mothers and pregnancies did not differ significantly between groups (Table 1).

**Table 1:** Characteristics of Mothers and Pregnancies N = 68.

	IVM	Control	p
	(n = 34)	(n = 34)	
Maternal Age (years). Mean (sd)	32.9 (3.3)	32.9 (3.3)	
Maternal BMI	22.6 (5.6)	22.3 (3.3)	0.78
Primipara (%)	83.3	79.3	0.69
Non-smoker (%)	100.0	91.2	0.09
Gestational Diabetes (%)	11.8	5.9	0.39
Fresh embryo transfer (%)	97.0	97.0	0.90
Gestational age (weeks). mean (sd)	39.1 (3.3)	39.3 (2.1)	0.74
Number of embryos transferred (%)			
* 1*	5.8	5.9	
* 2*	67.7	67.7	
* 3*	26.5	26.5	
Singletons (%)	88.2	88.2	
Delivery (%)			0.13
* Spontaneous delivery*	50.0	73.6	
* Induced delivery*	35.3	17.6	
* C-section before labor*	14.7	8.8	
Type of delivery (%)			0.13
* Vaginal*	64.7	81.2	
* C-section*	35.3	18.8	

Control : ICSI without IVM.

In both groups, mean maternal age was 32.9 years, mean gestational age was 39 weeks and 88% of pregnancies were singletons.

Overall, 11.8% of mothers in the IVM group were diagnosed with gestational diabetes, versus only 5.9% in the control group (*p* = 0.39) and 35.3% of IVM infants were born after Caesarean section, versus only 18.8% in the control group. However, none of these differences was significant. In addition, it is noticeable that maternal characteristics were comparable among women having given birth to boys or girls (data not shown).

The characteristics of infants at birth did not significantly differ between groups. However, the mean weight tended to be higher in the IVM group than in controls (3,120 g vs 2,967 g, p = 0.41) (Table 2).

**Table 2:** Characteristics of Infants at birth.

N = 76			
	IVM	control	P
	(n = 38)	(n = 38)	
Sex (% boys)	57.9	60.5	0.81
Birthweight (g). Mean (sd)	3119.5 (871.6)	2966.6 (742.7)	0.41
Height (cm). Mean (sd)	48.6 (4.7)	47.8 (3.5)	0.42
Head circumference (cm). Mean (sd)	33.6 (2.7)	33.7 (2.0)	0.86
Apgar ≥7 at 5 min (%)	91.7	97.2	0.30

Findings at one and two years are displayed in Table 3. The mean weight of the IVM group infants was significantly higher than that of the control group (10.3 kg vs 9.2 kg, *p*<0.001). IVM infants were also significantly taller than controls (76.8 cm vs 74.4 cm, *p*<0.01) and their BMI was greater (17.5 vs 16.5, p = 0.01).

**Table 3:** IVM & ICSI Outcomes at 1 year and two years.

	IVM	Control	p
At 1 year	(n = 38)	(n = 38)	
Weight (kg) Mean (sd)	10.3 (1.1)	9.2 (1.4)	0.0002
Height (cm). Mean (sd).	76.8 (4.0)	74.4 (3.2)	0.006
Head circumference (cm). Mean (sd)	46.9 (1.4)	46.4 (1.3)	0.08
BMI	17.5	16.6	0.01
**At 2 years**	**(n = 37)**	**(n = 37)**	
Weight (kg) at 2 year. Mean (sd)	12.9 (1.4)	12.0 (1.3)	0.004
Height (cm). Mean (sd).	87.2 (3.7)	86.8 (2.9)	0.62
Head circumference (cm). Mean (sd)	48.8 (1.6)	49.0 (2.3)	0.73
BMI	17.0	15.9	0.002

At the age of two years, IVM children were significantly heavier than controls (12.9 kg vs 12.0 kg, *p*<0.01), and their BMI was greater (17.0 vs15.9, p<0.01. However, there was no significant difference between groups in terms of height or head circumference.

Weight, height, head circumference and BMI were then studied separately for boys and girls at birth, and at one and two years of age (Table 4).

**Table 4:** IVM & ICSI weight, height and cranial circumference according to sex.

NT = 76						
	Boys			Girls		
	IVM	ICSI	p*	IVM	ICSI	p*
	22	23		16	15	
Birth						
Weight (g) at birth. mean (sd)	3034.5 (1015.5)	3139.6 (781.6)	0.80	3236.3 (636.4)	2701.3 (611.1)	0.03
range	540–4350	1020–4210		1880–4190	1580–3510	
Height (cm) at birth. mean (sd)	48.0 (5.8)	48.4 (4)	0.79	49.3 (2.4)	46.9 (2.5)	0.01
range	28–52	38–54		44–53	43–50	
Head circumference (cm) at birth. mean (sd)	33.3 (3.2)	34.3 (2.1)	0.21	34.1 (1.7)	32.9 (1.8)	0.04
range	22–37	29–37		30–37	30–37	
1 year						
Weight (kg) at 1 year. mean (sd)	10.4 (1.1)	9.6 (1.4)	0.12	10.2 (1.2)	8.6 (1.0)	<10^−3^
range	8.1–12.5	7.5–12.2		8.2–12.9	7.1–10.8	
Height (cm) at 1 year. mean (sd)	77.4 (4.0)	75.6 (3.1)	0.10	75.9 (3.9)	72.7 (2.5)	0.03
range	70–85	70–80		71–84	68–77	
BMI at 1 year. mean (sd)	17.4 (1.0)	16.8 (1.9)	0.22	17.6 (1.6)	16.2 (1.2)	0.01
range	15.8–19.1	13.7–20.5		14.9–20.8	14.4–18.5	
Head circumference (cm) at 1 year. mean (sd)	47.2 (1.4)	46.6 (1.2)	0.12	46.6 (1.4)	46.1 (1.3)	0.19
range	44–49.5	44–49		43–49	44–49	
2 years						
Weight (kg) at 2 years. mean (sd)	12.9 (1.3)	12.3 (1.3)	0.26	12.9 (1.5)	11.6 (1.2)	0.02
range	11.3–16.0	9.8–14.4		10.5–15.0	9.3–13.2	
Height (cm) at 2 years. mean (sd)	87.8 (3.9)	87.6 (2.3)	0.76	86.5 (3.3)	85.7 (3.3)	0.90
range	76–95	83–91		80–92	76–89	
BMI at 2 years. mean (sd)	16.8 (1.8)	16.0 (1.5)	0.27	17.2 (1.7)	15.8 (0.9)	0.02
range	13.6–21.1	12.1–18.2		15.1–20.3	14.5–17.2	
Head circumference (cm) at 2 years. mean (sd)	49.3 (1.3)	48.6 (1.7)	0.32	48.2 (1.8)	49.4 (2.9)	0.45
range	47–51	43–51		44–50	46–58	

*Comparison between IVM and ICSI. Kruskal Wallis test.

IVM girls were heavier than controls girls at birth, with a mean weight of 3,236 g vs 2,701 g respectively (*p* = 0.03). Similarly, birth heights of IVM girls were greater than those of control girls (49.3 cm vs 46.9 cm (*p* = 0.01)) and they also had a larger head circumference (34.1 cm vs 32.9 cm (*p* = 0.04)).

At the age of one year, IVM girls were still heavier (10.2 kg vs 8.6 kg (*p* = 0.001)), and taller (75.9 cm vs 72.7 cm (*p* = 0.03)) than the control girls and there was a significant difference in BMI (17.6 vs 16.2 (*p = *0.01)) between both groups of girls. However, the difference in head circumference was no longer significant.

At the age of two years, the sex effect remained, with IVM girls still heavier than control girls (12.9 kg vs 11.6 kg (*p* = 0.02)), and with a greater BMI (17.2 vs 15.8 (*p = *0.02)).

Regarding boys, no significant difference was underlined between the two groups for weight, height and head circumference either at birth, at one or two years of age.

## Discussion

We found no significant differences between IVM and control groups, in terms of maternal and obstetric characteristics. However, we highlighted a sexual dimorphism. Unlike boys, IVM girls had a greater weight, height and head circumference at birth than did control girls (ICSI). This difference between sexes was maintained over time, with IVM girls having a greater BMI than control girls at the ages of one and two years.

The follow-up rate was excellent, with 97% of the families completing the questionnaire when their children were two years old, thereby limiting selection bias.

The control group included infants born after ICSI. This choice of ICSI controls can be discussed. Most previous studies focusing on children conceived through IVM did not use control group [5], [6], [7]. In addition, comparative investigations [8], [9] mainly included infants conceived spontaneously. Since *in vitro* matured oocytes are fertilised by ICSI in the IVM protocol, the choice of an ICSI control group instead of natural pregnancies enabled a specific focus on the IVM technique itself. Furthermore the prevailing policy in our centre was to exclusively propose IVM to all women displaying PCOS criteria. Therefore it was not possible to recruit PCOS women having undergone ICSI after controlled ovarian hyperstimulation. Because of this, the results of the present investigation do not enable to precisely determine which part of the observed differences is due to the technique itself and to the underlying PCOS.

The sample size of this study (16 girls and 22 boys born after IVM and 15 girls and 23 boys in the control group) though limited, is similar to those of other studies [5], [8]. The statistical power of comparisons within sex was thus limited too. However, this limited power cannot induce a spurious significant difference between IVM and controls among girls, and it is important to point out that, with a similar sample size, differences were not significant among boys.

Women in the IVM group had a higher frequency of gestational diabetes (11.8% vs 5.9% (*p* = 0.39)), a higher frequency of Caesarean section (35.3% vs 18.8% (*p* = 0.3)), although neither of these differences were significant. Maternal mean BMIs were very similar in both groups (22.4 vs 22.1 kg/m^2^ (*p* = 0.78)). Soderstrom-Antilla *et al* [7] reported obstetric and perinatal outcomes in 43 women who had given birth to 46 infants after IVM. They reported a rate of gestational diabetes of 7% and a rate of delivery by Caesarean section of 35%. More recently, Buckett *et al.* [9] reported a significantly higher rate of gestational diabetes in women who became pregnant after IVM (17%) than in patients conceiving by ICSI (10%). This difference probably reflects the fact that all patients are suffering from PCOS, a risk factor for gestational diabetes, in the IVM population.

Indeed, previous studies have reported a higher risk of adverse pregnancy outcomes in the PCOS population [16], [17].

Boosma *et al*. [16] recently showed, in a meta-analysis, that women with PCOS were at higher risk of developing gestational diabetes, pregnancy-induced hypertension, preeclampsia and preterm births. More recently, Roos *et al*. [17] highlighted, in a population-based cohort study on singleton births based on the Swedish medical register between 1998–2007, that PCOS was also associated with preeclampsia, very preterm birth and an increase, by a factor of more than two, in the risk of gestational diabetes. Women with PCOS also had a higher risk of emergency and elective Caesarean section. The authors concluded that these risks of adverse pregnancy and birth outcome could not be explained by the reproductive technology used.

Even though the difference observed was not statistically significant (p = 0.41) we found a trend of slightly greater birth weight in the IVM group. This result is consistent with the findings of previous studies on children born from ICSI and IVM treatment [13] and children born after IVM and spontaneously conceived singletons [9].

However, one of the striking findings of our study was the difference in birth-weight, height and head circumference between IVM and control girls, but not in boys. This difference between sexes was maintained over time even after adjusting for gestational age and the exact age at examination.

No study of children born following IVM has, to our knowledge, ever investigated the outcome of these children according to sex [5] [6] [7] [8] [9]. We wondered whether the greater weight of IVM girls could be due to different distributions of singletons and multiples between the IVM and ICSI groups. Data showed that 81% of IVM girls were singletons compared to 67% of ICSI girls. After excluding twins, the difference between sexes remained.

Our results for IVM girls may be accounted for by adverse effects of the IVM technique, leading to sexual dimorphism and foetal programming process. However, they may also be accounted for by maternal characteristics.

Indeed, the frequency of gestational diabetes (with foetal hyperinsulinism) was higher in IVM than in control mothers. However, the frequency of diabetes is not sufficient in itself to account for the observed differences, particularly given the maintenance of these differences over time, even after the exclusion of diabetic mothers. Furthermore, gestational diabetes cannot account for the detection of these differences only in girls.

PCOS of mothers treated by IVM may also play a role. Roos *et al*. [17] reported that infants born to mothers with PCOS were more frequently large for gestational age, but other studies did not confirm this increase in the risk of macrosomia [18] [19] [20]. In a meta-analysis of pregnancy outcomes in women with PCOS, Boomsma *et al*. [16] did not confirm that infants born to mothers with PCOS had a greater birth weight. Indeed, in another study [21] the risk of being born small for gestational age was actually found to be greater in babies of mothers with PCOS. Beyond the potential impact of PCOS on growth, it is possible that certain treatments given to PCOS women during pregnancy may affect growth of the child [22]. These conflicting findings highlight the current lack of consensus regarding the impact of PCOS on birth weight.

It has been suggested that the higher level of exposure of males to prenatal androgens promotes foetal growth. Thus, as women with PCOS have higher plasma testosterone concentrations than other women, IVM girls may have been exposed to higher levels of testosterone, affecting their foetal growth and their birth weight, in particular.

Barry *et al.* [23] studied umbilical vein testosterone concentrations in female infants born to mothers with PCOS. They showed that these infants had higher androgen levels than boys or girls born to mothers who did not have this syndrome. We were unable to confirm this in our study because we had no data concerning testosterone levels in mothers and babies. Harriet *et al.* [24] compared birth weight in two situations affecting androgen exposure *in utero*: complete androgen insensitivity syndrome, in which the prenatal androgen response is impaired, and congenital adrenal hyperplasia, in which exposure to prenatal androgen is increased. They concluded that sex dimorphism in birth size was unrelated to prenatal androgen exposure. A recent study measuring the impact of prenatal exposure to high testosterone levels in sheep [25] found no difference in foetal weight between males and females.

In conclusion, our results for IVM girls are a matter for concern, but should be interpreted with caution. We cannot exclude a link with IVM technology, which may have different impacts on girls and boys. For example, IVM itself or the maternal serum used in IVM protocols may have a peri-conceptual effect on gene methylation, which may be altered in girls but not boys, leading to foetal programming.

However, it is not yet possible to exclude the possibility of a link to the underlying infertility of mothers with PCOS. It will therefore be important to continue following the outcomes of these infants, focusing, in particular, on girls conceived through IVM, to determine the potential impact of IVM on their future growth. It is also important to study the outcomes of girls conceived following IVM for which the indication was not linked to PCOS.
